# Noncoding RNAs and Cardiac Fibrosis

**DOI:** 10.31083/j.rcm2402063

**Published:** 2023-02-14

**Authors:** Changyong Wu, Suli Bao, Ruijie Li, Huang Sun, Yunzhu Peng

**Affiliations:** ^1^Department of Cardiology, The First Affiliated Hospital of Kunming Medical University, 650000 Kunming, Yunnan, China

**Keywords:** non-coding RNAs, myocardial fibrosis, biomarker, gene regulation, molecular mechanism

## Abstract

Myocardial fibrosis is a common pathological feature of various terminal cardiovascular diseases. Progressive fibrosis is the pathological basis for the development and progression of many cardiac arrhythmias and heart failure. There are no effective reversal drugs for myocardial fibrosis due to the lack of understanding of the molecular mechanisms. Noncoding RNAs, a class of RNAs that do not function in coding proteins, have been found to be intimately involved in the life cycle of cardiomyocyte differentiation, transcription and apoptosis and are important regulators of cardiovascular disease. An increasing number of studies have shown that noncoding RNAs regulate the proliferation and transformation of cardiac fibroblasts through related signaling pathways and can be used as potential biomarkers and novel therapeutic targets for cardiac fibrosis. This article reviews the relationship between noncoding RNAs and cardiac fibrosis.

## 1. Introduction

Myocardial fibrosis is a common maladaptive pathological change in multiple advanced cardiovascular diseases in response to cardiac pressure or volume overload, which promotes the proliferation and activation of cardiac fibroblasts (CFs) and excessive extracellular matrix (ECM) production within the myocardium. Excessive synthies of multiple cytokines, growth factors and chemokines can alter CF biological activity, leading to cardiac remodeling that results in cardiac dysfunction, conduction abnormalities and reduced compliance, ultimately leading to arrhythmia and heart failure (HF).

**Fig. 1.**
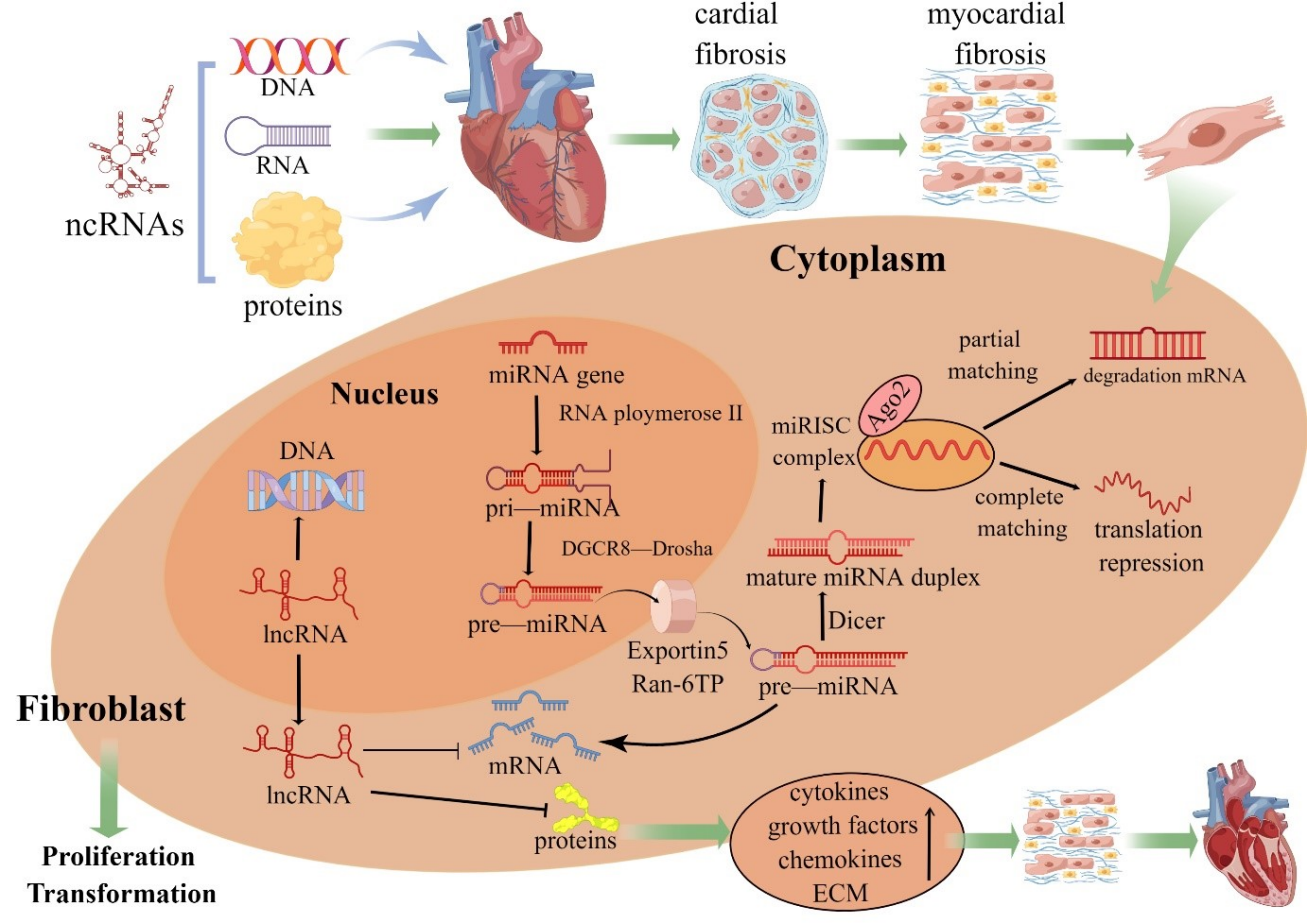
**ncRNAs in fibroblast biology**. Pri-miRNAs are processed by Drosha into pre-miRNAs before the endonuclease Dicer generates a mature miRNA. Functional miRNAs are ultimately coupled to Argonaute 2 protein and then incorporated into the RNA-induced silencing complex. When a miRNA and target mRNA are completely complementary, the target mRNA is degraded; when two sequences are partially complementary, the specific gene is silenced through translational suppression of the target mRNA. The mechanism of mRNA regulation by miRNAs depends on the degree of sequence complementarity between the miRNA and 3´UTR motif in the target mRNA gene. One miRNA sequence can target different mRNAs, and the expression of one mRNA sequence can be downregulated by different miRNAs. In contrast, lncRNAs are involved in the proliferation and transformation of cardiac fibroblasts through complementary interactions with DNA, miRNA sponges and indirect or direct regulation of proteins. MiRNAs and lncRNAs are important regulators of intracellular gene expression, and excess synthesis of multiple cytokines, growth factors and chemokines alters the biological activity of CFs, resulting in increased extracellular matrix (ECM) synthesis that leads to cardiac remodeling (By Figdraw).

Most sequences in the human genome do not encode proteins, and only 1.5–2% of the genome is capable of being transcribed into RNA that encodes proteins [1]. With 200 nucleotides as the maximum length, noncoding RNAs (ncRNAs) are divided into long-chain noncoding RNAs (lncRNAs) and short-chain noncoding RNAs, of which microRNAs (miRNAs) are the most characteristic ncRNAs. In addition, there is a nonlinear ncRNA called circular RNA (circRNA). Recent studies have confirmed that ncRNAs are important regulators of cardiovascular diseases and are involved in the life cycle of cardiomyocyte differentiation, transcription and apoptosis [2] (Fig. 1). CFs are important components of cardiomyocytes with diverse origins, and ncRNAs are differentially expressed in myocardial fibrotic tissues. Therefore, exploring the pathophysiological functions and mechanism of ncRNAs in cardiovascular disease-induced myocardial fibrosis can provide new strategies for the diagnosis, treatment and prognosis of cardiovascular diseases. This review discusses the relationship between noncoding RNAs and cardiac fibrosis.

## 2. MiRNAs in Cardiac Fibrosis

MiRNAs are single stranded RNAs molecules about 22 nucleotides in length that can regulate gene expression at the post-transcriptional level and play a critical role in cardiac fibrosis, mainly including left ventricular fibrosis, right ventricular fibrosis and atrial fibrosis (Table 1, Ref. [3, 4, 5, 6, 7, 8, 9, 10, 11, 12, 13, 14, 15, 16, 17, 18, 19, 20, 21, 22, 23, 24, 25, 26, 27, 28, 29, 30, 31, 32, 33, 34, 35, 36, 37]).

**Table 1.** **MiRNAs in cardiac fibrosis**.

miRNA	Stimulation	Target gene	Function	Reference
miR-10a	AF	TGF-β1/Smad	Anti	[27]
miR-20a-5p	DCM	ROCK2, JNK/NF-kB	Anti	[11]
miR-21	HF, DCM, TAC, AF	WWP-1, TGF-β1/Smad2	Anti	[9, 13, 21]
miR-21-3p	DM	FGFR1,FGF21,PPARγ	Anti	[28]
miR-21-5p	VMC, ARVC	TNFα, IL-6, Vnt, Hippo	Anti	[16, 31]
miR-27a-5p	TAC	Egr3	Anti	[14]
miR-29	MI	PI3K/mTOR/HIF-1α/VEGF	Anti	[6]
miR-29a-3p	PAH	THBS2	Anti	[17]
miR-29b	TAC, AS, AF	TGF-β/Smad, ALK5	Anti	[15, 25]
miR-30c	AF	TGF-β2	Anti	[26]
miR-30d	HF	ITGA5	Anti	[7]
miR-34a	Age, AMI, DOX	PNUTS, *Bcl-2*, *SIRT1*	Anti	[20, 32]
miR-101	MI	*RUNX1*/TGF-β1/Smad	Anti	[5]
miR-129-5p	HF	Smurf1/PTEN	Anti	[8]
miR-132	AF, Ang II	CTGF	Anti	[23]
miR-133b	DOX, HT	PTBP1, TAGLN2	Anti	[33, 34]
miR-135b	ARVC	Vnt, Hippo	Anti	[16]
miR-146b-5p	AF, MI	TIMP/MMP9	Anti	[30]
miR-155	MI, VMC		Anti	[4, 35]
miR-195	HBP	TGF-β1/Smad3	Anti	[10]
miR-205	AF	P4Hα3, JNK	Anti	[29]
miR-325-3p	PAH	HE4, PI3K/AKT	Anti	[18]
miR-495	DOX	AKT	Anti	[36]
miR-4443	AF	THBS1, TGF-β1/α-SMA	Anti	[24]
miR-1	MI	UPS	Pro	[3]
miR-23b-3p	AF	TGF-β3/Smad3	Pro	[22]
miR-27b-3p	AF	TGF-β3/Smad3	Pro	[22]
miR-148-3p	PAH	HIF-1α, DNMT1	Pro	[19]
miR-340-5p	DCM	*Mcl-1*	Pro	[12]
miR-1468-3p	Age	TGF-β1/p38	Pro	[37]

*Bcl-2*, B lymphocytoma-2; DNMT1, DNA methyltransferase 1; FGFR1, fibroblast growth factor receptor 1; HBP, high blood pressure; HE4, human epididymis protein 4; HIF-1α, hypoxia inducible factor 1α; MMP9, matrix metalloproteinase 9; *Mcl-1*, myeloid leukemia-1; P4Hα3, prolyl-hydroxylase α polypeptide III; PPARγ, peroxisome proliferator-activated receptor γ; PTBP1, poly-pyrimidine bundle-binding protein 1; *RUNX1*, runt-related transcription factor 1; *SIRT1*, sirtuin 1; TAGLN2, transgelin 2; TIMP, tissue inhibitor of metalloproteinase; WWP-1, WW structural domain-binding protein-1.

### 2.1 Left Ventricular Fibrosis

An increasing number of studies have shown that miRNAs participate in the development of myocardial fibrosis, but their functions and mechanisms remain to be fully elucidated [38]. Myocardial infarction (MI) is the leading cause of death from cardiovascular disease. The myocardium slowly enters a long-term progressive fibrotic process after the acute phase of infarction, which is an adaptive remodeling in the early stage, but the persistent fibrotic response accelerates HF after MI. MiRNAs were significantly differentially expressed in MI, with miR-1 expression levels increasing after MI. Inhibiting miR-1 expression through the use of a miR-1 antagomir significantly reduced left ventricular end-diastolic internal diameter, collagen proliferation and TGF-β expression after MI but increased ejection fraction and improved myocardial fibrosis and cardiac functions [3]. However, miR-155 exerted no marked effect on left ventricular volume, left ventricular mass or ejection fSSraction, although the myofibroblast density was obviously lower than that in the control [4]. *RUNX1*, known as acute myeloid leukemia 1 (*AML 1*), is a transcription factor with a highly conserved protein sequence and is involved in the expression of genes related to cell differentiation and proliferation. MiR-101 targeted *RUNX1*, and therefore, overexpression of miR-101 or silencing of *RUNX1* might decrease infarct size, attenuate myocardial fibrosis and inhibit apoptosis, thereby improving cardiac functions. Moreover, miR-101 played a protective role against cardiac remodeling via inactivation of the *RUNX1*-dependent transforming growth factor β1/Smad family member 2 (TGF-β1/Smad2) signaling pathway [5]. In addition, inhibition of miR-29 expression activated the PI3K/mTOR/HIF-1α/VEGF pathway to promote microangiogenesis and reduce myocardial fibrosis after MI [6].

Post-MI myocardial interstitial ischemic edema can decrease myocardial compliance and contractility, increase left ventricular end-diastolic pressure and volume, and eventually progression to HF or even death. MiR-30d inhibited fibroblast proliferation and activation by directly targeting integrin α5 to enhance cardiac functions in the acute phase of ischemic HF in mice [7]. Myocardial structure, cardiac functions and oxidative stress were enhanced, and fibrosis in myocardial tissue was reduced after Smad ubiquitin regulatory factor 1 (Smurf1) knockdown. MiR-129-5p targets Smurf1, represses the ubiquitination of phosphatase and tensin homolog (PTEN), and promotes PTEN expression, which attenuates the cardiac functions of chronic HF rats [8]. Hinkel *et al*. [9] provided the first evidence showing the feasibility and therapeutic efficacy of miR-21 inhibition in a large animal model of HF and found that silencing miR-21 reduced cardiac fibrosis and hypertrophy to improve cardiac function at 33 days after ischemia reperfusion injury.

In the early stage of cardiovascular diseases, such as chronic diseases, degenerative valve diseases and cardiomyopathies, left ventricular wall thickening and myocardial fibrosis progression result in reduced cardiac compliance and function, which leads to left ventricular enlargement and the development of left heart failure in the late stage. Studies have shown that miRNAs are also involved in left ventricular fibrosis due to these diseases [39]. For example, the miR-195 expression level was remarkedly reduced in hypertensive rats, with disorganized myocardial cells, thickened myocardial fibers and myocardial fibrosis in a hypertension group compared to controls. Overexpression of miR-195 inhibited TGF-β1/Smad3 signaling pathway activity and related molecules, further repressing myocardial fibrosis [10]. Diabetic cardiomyopathy (DCM) is initially characterized by early diastolic dysfunction, left ventricular remodeling, hypertrophy and myocardial fibrosis. Well-characterized miRNAs involved in the development of DCM including miR-20a-5p, miR-21 and miR-340-5p [11, 12]. MiR-340-5p expression has been found to be dramatically increased in the heart tissue of mice and cardiomyocytes under diabetic conditions, and its overexpression exacerbated the apoptosis of cardiomyocytes, elevated reactive oxygen species production and impaired mitochondrial function, leading to extensive cardiac fibrosis and severe dysfunction. Later studies revealed that miR-340-5p caused severe cardiac dysfunction by repressing the expression of the target gene myeloid leukemia-1 (*Mcl-1*), which suggested that miR-340-5p plays a crucial role in the development of DCM and can be targeted for therapeutic intervention [12]. In addition, other miRNAs, such as miR-21, miR-27a-5p and miR-29b, play vital roles in pressure-load induced cardiac fibrosis caused by transverse aortic constriction (TAC) [13, 14, 15]. García *et al*. [15] assessed the condition of 103 patients with aortic stenosis preoperatively and one year after aortic valve replacement surgery and found that the preoperative plasma expression of miR-29b paralleled the severity of hypertrophy and was a significant negative predictor of reverse remodeling after aortic valve replacement surgery, indicating that the miR-29b level may be a potential prognostic biomarker. Thottakara *et al*. [40] first reported that miR-4454 expression was significantly elevated in patients with hypertrophic cardiomyopathy and was correlated with the severity of myocardial fibrosis, as determined through comparison cardiac magnetic resonance, suggesting that miR-4454 may be a potential biomarker of fibrosis. Multiple miRNAs are involved in the process of myocardial fibrosis, indicating that miRNAs are important regulators of myocardial fibrosis and might be potential therapeutic targets.

### 2.2 Right Ventricular Fibrosis

The right ventricle is under volume or pressure overload due to left heart failure, heart valve diseases, pulmonary artery hypertension (PAH) and other diseases, which leads to the formation of right ventricular fibrosis, and scar tissue causes right ventricular dysfunction. In PAH, sustained pressure overload exerts mechanical stress on the right ventricular interstitium and CFs, which increases collagen production by releasing TGF-β to promote proliferative activation of CFs as well as increasing mRNA expression and upregulating α-smooth muscle actin activity (α-SMA). In the early stage of the disease, increasing collagen can support the myocardium in withstanding high pressure to maintain the right ventricular structure. However, with the progression of disease, adaptive myocardial collagen accumulation follows maladaptive changes in collagen network structure and loss of ECM integrity, eventually leading to decreased cardiac compliance, cardiac dysfunction and arrhythmic events [41], such as increasing right ventricular wall stiffness and filling pressure indirectly induces supraventricular arrhythmias though transmission to the right atrium. In addition, arrhythmogenic right ventricular cardiomyopathy (ARVC) is an inherited cardiomyopathy characterized by progressive fibro-fatty replacement of right ventricular myocardial, arrhythmias and risk of sudden death. Currently, the pathophysiological role of miRNAs in ARVC has not been fully elucidated. For example, the expression of miR-21-5p, miR-135b and miR-185-5p was found to be distinctly elevated and to regulate myocardial fatty fibrosis via the Wnt and Hippo pathways in patients with ARVC [16, 42].

Hsu *et al*. [17] found that the levels of circulating miR-29a-3p and thrombospondin-2 (THBS2) decreased and increased, respectively, in mice and patients with PAH. MiR-29a-3p directly targets and regulates THBS2 expression to inhibit the proliferation of CFs, which exerts a direct antifibrotic effect on PAH-induced cardiac fibrosis. MiR-325-3p targets and regulates human epididymis protein 4 to activate the PI3K/AKT signaling pathway, and the action of this miRNA was found to inhibit CF transformation and attenuate right ventricular fibrosis in PAH rats [18]. A mitochondrial metabolism study of PAH-induced right ventricular fibrosis revealed that increased pyruvate dehydrogenase activity inhibited mitochondrial superoxide dismutase 2 (SOD2) and $H_{2}$$O_{2}$ production; activated hypoxia-inducible factor-1α (HIF-1α); increased pyruvate dehydrogenase kinase isoforms 1 and 3, TGF-β1 and CTGF expression; increased CF proliferation and collagen production; promoted fibrosis formation and reduced right ventricular function. Among these outcomes, HIF-1α activation, in particular, reflects increased DNA methyltransferase 1 expression, which has been associated with a decrease in the regulatory effector of miR-148-3p [19]. Notably, miRNA distribution has been reported to differ between the right and left ventricles in mammals, and the functional mechanisms of right ventricular fibrosis are less clear than those of the left ventricle. Boon *et al*. [20] showed that miR-34a was induced in the aging heart and that *in vivo* silencing or gene deletion reduced age-related apoptosis of cardiomyocytes. MiR-34a-targeting PNUTS induced the DNA damage response and telomere shortening to improve functional recovery after acute myocardial infarction (AMI) [20]. Whether a miRNA-based therapeutic strategy to prevent left ventricular fibrosis is effective on the right ventricle remains unknown, and the miRNAs to target have yet to be determined.

### 2.3 Atrial Fibrosis

Atrial fibrillation (AF) is the most common cardiac arrhythmia and a result of atrial remodeling. Atrial remodeling, characterized by persistent biventricular enlargement and fibrosis of myocardial tissue, is caused by atrial volume or pressure overload and eventually progresses to diastolic insufficiency or even sudden death by thromboembolism. MiRNAs are significantly differentially expressed in AF. For example, miR-21, miR-23b-3p and miR-27b-3p expression has been found to be significantly increased in atrial tissue of patients with AF [21, 22, 43], but the levels of miR-132 and miR-443 were decreased [23, 24]. The main target of miR-21 is TGF-β. The results of an experimental study performed with human fibroblasts by Tao *et al*. [21] confirmed that downregulated miR-21 increased the expression of WW structural domain-blinding protein-1 to inactivate the TGF-β1/Smad2 signaling pathway, inhibit the proliferation of CFs, and reduce collagen I and III levels and the collagen volume fraction. Importantly, miR-29b, miR-30c and miR-10a also regulate the TGF-β/Smad pathway, suggesting that miRNAs may be potential therapeutic targets for the prevention of atrial fibrosis [25, 26, 27]. Pan *et al*. [28] established a coculture model with atrial fibroblasts and adipocytes and found that miR-21-3p regulated FGFR1, FGF21, and PPARγ to control epicardial adipocyte brownout (EAT) and ameliorate glucose-induced atrial fibrosis, suggesting that modulating EAT may be a new strategy to prevent or treat atrial fibrosis or AF in DCM. Reducing miR-205 expression in AF rats attenuated atrial fibrosis by targeting prolyl-hydroxylase α polypeptide III (P4Hα3) to inhibit CF proliferation and migration and inactivating the JNK pathway [29]. Other miRNAs are also involved in atrial remodeling, including miR-133, miR-590 and miR-146b-5p, and all were downregulated in a canine model of AF [30, 44]. The expression of miR-146b-5p, whose target gene is TIMPs, was upregulated in atrial cardiomyocytes during AF. The inhibition of miR-146b-5p expression inhibited the acquisition of a cardiac fibrosis phenotype in MI mouse models. The expression of fibrotic markers MMP9, TGF-β1 and COL1A1 was significantly downregulated, while that of IMP4 was significantly upregulated by miR-146b-5p inhibition in the cardiomyocytes of MI hearts. In contrast, *in vitro*, miR-146b-5p regulated TIMP/MMP9-mediated ECM synthesis, increasing collagen synthesis in a human-induced pluripotent stem cell-derived atrial cardiomyocyte fibroblast coculture cell model [30].

## 3. LncRNAs in Cardiac Fibrosis

**Fig. 2.**
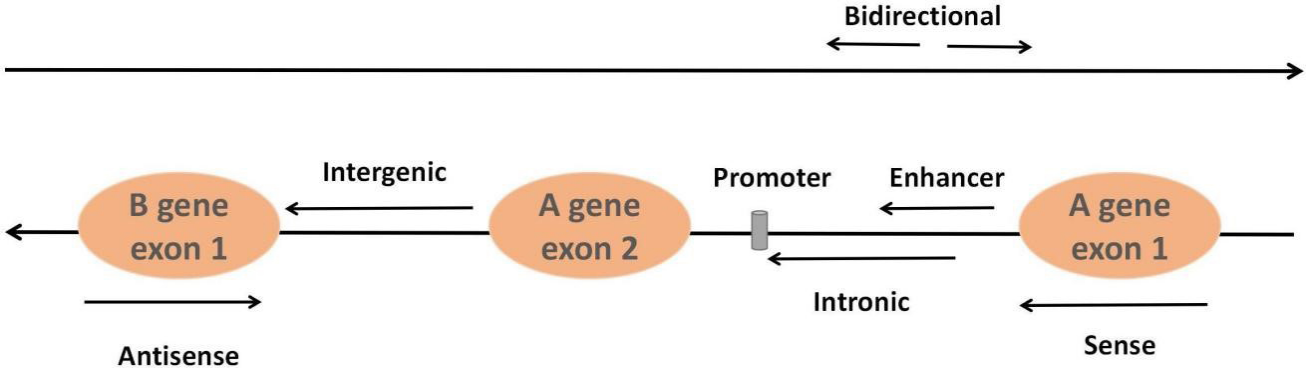
**Classification of lncRNAs**. Sense lncRNAs are transcribed from the sense coding chain with exons, and they usually cover or overlap with protein-coding genes. In contrast, antisense lncRNAs are transcribed from genes encoding antisense proteins. Intergenic lncRNAs refer to lncRNAs in the genomic interval between two genes. Intronic lncRNAs are formed from the introns of second transcripts. Bidirectional lncRNAs are transcribed from the opposite direction and spaced approximately 1 kb apart. Enhancer lncRNAs originate from enhancer regions in protein-coding genes.

LncRNAs, accounting for approximately 80%–90% of all ncRNAs, constitute a class of ncRNAs more than 200 nucleotides in length. Most lncRNAs are transcribed by RNA polymerase II and modify both ends of the seven-methylguanosine triphosphate cap at the 5´ end and the polyadenylate tail at the 3´ end by utilizing the same splicing signal as the coding gene. They can be classified into six types based on their relative position in the genome of neighboring coding regions: sense lncRNAs, antisense lncRNAs, intergenic lncRNAs, intronic lncRNAs, enhancer lncRNAs and bidirectional lncRNAs [45] (Fig. 2). The classical mechanisms of lncRNAs can be divided into four categories: signal, decoy, guide and scaffold [46]. Signal: lncRNAs not only regulate neighboring genes in cis conformation but also play a trans-regulatory role in the expression of genes that are not closely related to their transcription sites. Decoy: the lncRNA-miRNA-mRNA axis, and lncRNAs adsorb and inhibit miRNAs to regulate mRNA molecular functions through molecular sponge action. Guidance: lncRNAs can recruit and bind related proteins through molecular interactions and guide complexes to specific targets. Scaffolding: lncRNAs interact with chromatin modification complex components or proteins such as transcription factors to bind to molecular scaffolds that influence gene expression. The advantages of lncRNAs that can be used as potential biomarkers include high stability and detectability, accurate quantification by highly sensitive methods such as real-time PCR and the fact that changes in the levels of lncRNAs may reflect the underlying mechanisms of disease.

In recent years, significant progress has been made in the study of lncRNAs regulating cardiac fibrosis (Table 2, Ref. [47, 48, 49, 50, 51, 52, 53, 54, 55, 56, 57, 58, 59, 60, 61, 62]). Zhang *et al*. [47] demonstrated that the lncRNA H19 level was significantly downregulated in mice with MI. Functionally, enforced H19 expression dramatically reduced infarct size and improved cardiac functions by mitigating myocardial apoptosis and decreasing inflammation. Mechanistically, H19 regulated the expression of KDM3A to ameliorate MI-induced myocardial injury in a miR-22-3p-dependent manner. The expression level of the lncRNA MHRT was increased in mice with MI or cardiac fibrosis and treated with TGF-β1. MHRT promoted cardiac fibrosis by inhibiting miR-3185 and increasing myocardial collagen deposition and CF proliferation [48]. In contrast, lncRNA 554 was mainly expressed in CFs, and knocking down lncRNA 554 inhibited CF migration and ECM deposition [49]. LncRNA Gpr19 reduced oxygen glucose deprivation/recovery induced left ventricular cardiomyocytes of MI mice exposed to miR-324-5p and mitochondrial fission regulator 1 by regulating oxidative stress and apoptosis and attenuating scar formation in myocardial fibrosis [50]. In addition to, the lncRNA TUG1/miR-133b/CTGF and lncRNA XIST/miR-155-5p pathways, the lncRNA Wisper and other signaling axes were confirmed to regulate myocardial fibrosis after MI [51, 52, 53].

**Table 2.** **LncRNAs in cardiac fibrosis**.

LncRNA	Stimulation	Target gene	Function	Reference
H19	MI, AF	miR-22-3p/KDM3A	Anti	[47, 56]
		miR-29a/b-3p, VEGFA		
554	MI	TGF-β1	Anti	[49]
Gpr19	MI	miR-324-5p, Mtfr1	Anti	[50]
Wisper	MI	TIAI	Anti	[53]
NRON	Ang II	miR-23a	Anti	[57]
Chast	TAC, AS	Pleckstin	Anti	[59]
SOX2OT	HF	TGF-β1/Smad	Anti	[60]
KCNQ1OT1	DOX	FUS	Anti	[61]
MHRT	TGF-β1	miR-3185	Pro	[48]
TUG1	MI	miR-133b/CTGF	Pro	[51]
XIST	AMI	miR-155-5p	Pro	[52]
PVT1	AF	miR-128-3p/TGF-β1/Smad	Pro	[54]
NEAT1	AF	miR-320/NPAS2	Pro	[55]
MALAT1	HBP	SMA	Pro	[58]
ROR	VMC	*C-myc*, IL-6	Pro	[62]

FUS, fusion-type sarcoma; Gpr19, G protein-coupled receptor 19; KCNQ1OT, KCNQ1 opposite strand/antisense transcript 1; KDM3A, lysine-specific demethylase 3A; MALAT1, Metastasis-associated lung adenocarcinoma transcript 1; Mtfr1, mitochondrial fission regulator 1 Antibody; NEAT1, nuclear-enriched abundant transcript 1; PVT1, plasmacytoma variant translocation 1; TUG1, taurine upregulation gene 1; XIST, X-inactive specific transcript; Wisper, Wisp2 super-enhancer-associated RNA.

LncRNAs, important regulators of atrial fibrosis that can increase CF proliferation and collagen expression leading to excessive RCM deposition, can promote the formation of atrial fibrosis in AF by competing with endogenous miRNAs. These lncRNAs have been found in pathways and are involved in the regulation of atrial fibrosis. For example, the lncRNA PVT1 and the lncRNA NEAT1 were increased in atrial muscle tissue of AF patients and positively correlated with collagen I and III [54, 55]. PVT1 overexpression facilitated TGF-β1/Smad signaling activation *in vitro* assay and acted as a sponge for miR-128-3p to facilitate Sp1 expression, which activated the TGF-β1/Smad signaling pathway *in vivo* and promoted atrial fibrosis by increasing Ang II-induced atrial fibroblast proliferation and collagen production [54]. However, NEAT1 could negatively regulate miR-320 expression by acting as a competitive endogenous RNA (ceRNA). MiR-320 directly targetes NPAS2 and suppresses its expression in CFs, which attenuates Ang II-induced atrial fibroblast proliferation, migration, and collagen production [55]. The level of plasma lncRNA H19 was significantly elevated in patients with AF, while miR-29a-3p and miR-29b-3p were markedly decreased [56]. Upregulation of H19 expression and downregulation of miR-29a/b-3p expression facilitated proliferation and synthesis of ECM-related proteins, but si-VEGFA was able to reverse the promotion of miR-29a/b-3p on proliferation of CFs and ECM-related protein synthesis [56]. In contrast, the lncRNA NRON promoted M2 macrophage (M2M) polarization and attenuated atrial fibrosis by inhibiting exosomal miR-23a in atrial myocytes [57].

In addition, lncRNAs play important regulatory roles in other diseases. For example, Li *et al*. [58] found that overexpression of the lncRNA MALAT1 increased arterial smooth muscle cell activity and caused severe myocardial fibrosis in spontaneously hypertensive rats. The lncRNA Chast was specifically upregulated in hypertrophic myocardial tissue of mice with aortic coarctation and patients with aortic stenosis and negatively regulated protein family M member one of the pleckstrin homologous structural domains (in the strand opposite to that carrying Chast), impeding cardiomyocyte autophagy and hypertrophy, suggesting that Chast may be a potential target for preventing cardiac remodeling [59]. HF is the end-stage manifestation of cardiovascular disease. SOX2OT knockdown reduced myocardial injury and collagen in HF mice, and the expression of collagen I, β-SMA, TGF-β1 and p-Smad3 was inhibited after SOX2OT downregulation in HF mice and ISO-induced CFs. SOX2OT promoted myocardial fibrosis in HF by activating TGF-β1/Smad3, and Smad3 then interacted with the SOX2OT promoter to form a positive feedback loop [60].

## 4. CircRNAs in Cardiac Fibrosis

CircRNAs are novel endogenous ncRNAs that show high conservation and stability. CircRNAs can be divided into three categories depending on the source: intron (circular intronic RNA, ciRNA); exon (exonic circular, ecRNA); and exon and intron (exon–intron circular RNA, EIciRNA). Target genes are regulated by sponging miRNAs, interacting with proteins and regulating the degradation and stability of mRNAs. An increasing number of studies have reported that circRNAs are emerging as regulators of pathophysiology in many diseases. However, the expression and function of circRNAs in cardiac fibrosis remain largely unknown.

Zhang *et al*. [63] found that in inducing CF activation with TGF-β1 or AngII significantly inhibited the expression of the circRNA circ-BMP2K and miR-455-3p but promoted the expression of SUMO1. Notably, circ-BMP2K downregulated SUMO1 expression by sponging miR-455-3p, which ultimately inhibited the activation, growth and migration of CFs. Sun *et al*. [64] found that overexpression of circ-LAS1 L promoted SFRP5 expression and inhibited α-SMA, type I and type III collagen expression, thereby inhibiting the proliferation and migration of CFs. Later studies showed that circ-LAS1 L regulates the biological properties of CFs by targeting the miR-125b/SFRP5 axis. Wang *et al*. [65] showed that M2 M-derived small extracellular vesicles containing circ-Ube3a promoted proliferation, migration and CFs by directly targeting the miR-138-5p/Rhoc signaling axis and promoting phenotypic transformation and exacerbating myocardial fibrosis after AMI. However, Li *et al*. [66] found that Ang II promoted the activation, proliferation and migration of CFs mediated via the circ-CELF1/miR-636/DKK2 signaling axis, and both miR-636 inhibitors and DKK2 were effective in attenuating myocardial fibrosis and enhancing cardiac functions in AMI mice. Garikipati *et al*. [67] found that in the heart tissue of MI mice and patients with ischemic cardiomyopathy, circ-Fndc3b expression was reduced, and overexpression of circ-Fndc3b exerted cardioprotective effects by promoting myocardial infarct zone neovascularization and reducing fibrosis in the infarct zone. Further studies revealed that circ-Fndc3b interacted with the FUS protein to regulate VEGF expression and signaling, thereby reducing myocardial fibrosis after myocardial infarction (Table 3, Ref. [63, 64, 65, 66, 67, 68, 69, 70, 71, 72]).

**Table 3.** **CircRNA in cardiac fibrosis**.

CircRNA	Stimulation	Target gene	Function	Reference
BMP2k	TGF-β1/Ang II	miR-455-3p/SUMO1	Anti	[63]
LAS1L	AMI	miR-125b/SFRP5	Anti	[64]
CELF1	AMI	miR-636/DKK2	Anti	[66]
Fndc3b	MI	FUS	Anti	[67]
Yap	TAC	TPM4, ACTG	Anti	[68]
NIgn	DOX	H2AX	Anti	[69]
Foxo3	HT	miR-433, miR-136	Anti	[70]
FSCN1	HT	tDCs	Anti	[71]
Ube3a	AMI	miR-138-5p/Rhoc	Pro	[65]
000203	Ang II	miR-26b-5p	Pro	[72]

BMP2K, BMP-2 inducible kinase; CELF1, gugbp Eeav-like family member1; Foxo3, forkhead box O3; FSCN1, fascin actinbundling protein 1; Fndc3b, fibronectin type III domain-containing protein 3B; LAS1L, LAS1-like; SUMO1, small ubiquitin-like modifier-1; SFRP5, secreted frizzled related protein 5; tDCs, tolerogenic dendritic cells; TPM4, tropomyosin-4; Ube3a, ubiquitin protein ligase E3A; Yap, Yes-associated protein.

## 5. NcRNAs and Other Factors Associated with Cardiac Fibrosis

### 5.1 Aged-Associated Fibrosis

Age exacerbates mortality from cardiovascular disease in elderly individuals. During cardiac aging, the accumulation of senescent cells and the deposition of ECM with collagen lead to a progressive decline in cardiac functions. A 3% increase in ECM volume and a 50% increase in the risk of all-cause mortality have been reported [73]. Studies have shown that ncRNA expression correlates with aging. For example, miR-1468-3p expression has been shown to be increased in healthy elderly hearts and to promote cardiac fibrosis by enhancing TGF-β1/p38 signaling [37]. In MI mouse models and heart biopsy samples from patients with aortic stenosis, the lncRNA Wisper was found to be associated with cardiac fibrosis, with Wisper regulating the CF gene expression program, and to be essential for the proliferation, migration and survival of CFs [53]. CircRNA-Yap inhibited cardiac fibrosis by interacting with tropomyosin-4 and γ-globular actin interactions, inhibiting actin polymerization and subsequent fibrosis [68]. CircRNA-000203 enhanced the expression of three fibrosis-related genes, COL1A2, COL3A1 and α-SMA, by inhibiting miR-26b-5p expression in mouse CFs [72]. Multiple ncRNAs are regulated in the aging heart and may serve as potential targets for age-related cardiac fibrosis therapy.

### 5.2 Myocarditis-Associated Fibrosis

Viral myocarditis (VMC) is local or diffuse damage to the myocardial parenchyma or interstitium caused directly by viral infection or indirectly by immune system dysfunction and is accompanied by cardiomyocyte destruction, reparative fibrosis and ultimately HF. NcRNAs regulate the viral life cycle and immune and inflammatory responses by targeting viral or host genes. MiR-155, lncRNA AK085865 and lncRNA MEG3 regulated macrophage polarization and reduced myocardial injury, which was correlated with a reduction in the development of myocardial fibrosis [35, 74, 75]. Studies in mice with autoimmune myocarditis revealed that silencing miR-21a-5p resulted in a significant reduction in TNFα, IL-6 and collagen I expression, reducing excessive infiltration of damaging myocardial cells and inhibiting myocardial fibrosis formation [31]. In an isoproterenol-induced myocardial fibrosis model of VMC, the lncRNA ROR upregulated *c-myc* expression and increased serum IL-6 levels, thereby facilitating the proliferation and differentiation of CFs. This suggested that lncRNA ROR plays an important role in chronic VMC [62]. Differential expression of circRNAs has been found in patients with fulminant myocarditis and in mice, and a bioinformatics analysis revealed the involvement of severely dysfunctional immune signaling pathways, including TNF and T-cell receptors, in VMC [76, 77]. Compared to miRNAs, other lncRNAs and circRNAs, ncRNAs have not been studied in detail in the field of mechanistic development of VMC and may be potential biomarkers and therapeutic targets for VMC in the future.

### 5.3 Drug-Associated Fibrosis

In recent decades, the mechanisms of drug-induced cardiotoxicity, mainly DNA damage, excessive reactive oxygen species production, mitochondrial dysfunction, endoplasmic reticulum-mediated apoptosis, and disruption of calcium homeostasis have been studied. With the deepening of research, it has been shown that ncRNAs play key roles in the mechanisms of cardiotoxicity induced by drugs such as doxorubicin (DOX) [78, 79]. For example, silencing miR-34a upregulated B lymphocytoma-2, and sirtuin 1 attenuated DOX-induced cardiotoxicity, reducing the apoptosis rate, attenuating the inflammatory response, and inhibiting senescence and fibrosis in rats [32]. MiR-133b attenuated polypyrimidine tract bundle-binding protein 1 and transgelin 2 to regulate apoptosis and mediate cardiac fibrosis induced by adriamycin, implying that miR-133b may be a potential biomarker of adriamycin-induced cardiac injury [33]. Endogenous miR-495-3p protects cells against DOX-induced cardiotoxicity by activating the AKT pathway *in vivo* and *in vitro * [36]. In addition, the expression of the lncRNA KCNQ1OT1 downregulated fusion-type sarcoma in oocytes and reduced the myocardial fibrosis area in DOX-treated mouse models [61]. Increasing the expression of circ-Nlgn decreased cardiac function and induced cardiac fibrosis by upregulating Gadd45b, Sema4C and RAD50 and activating p38 and pJNK in circNlgn transgenic mouse hearts. Silencing circ-NIgn prevented DOX-induced expression of fibrosis-associated molecules. The Nlgn173 protein translated by circ-NIgn can bind and activate H2AX and the production of γH2AX, which results in the upregulation of IL-1b, IL-2Rb, IL-6, EGR1 and EGR3. Later studies showed that silencing these molecules in the signaling pathway could prevent cardiomyocyte apoptosis, reduce CF proliferation and inhibit collagen production, mitigating the side effects of DOX in cancer patient treatment [69].

### 5.4 Transplantation-Associated Fibrosis

Heart transplantation (HT) is the best treatment option for end-stage HF. However, HT can cause cardiac ischemia reperfusion injury. In a mouse model of ectopic HT, 59 miRNAs were dysregulated in transplanted hearts with ischemia reperfusion injury compared to undamaged transplanted hearts [80]. In contrast, circ-Foxo3 is a newly discovered molecular regulator that protects cardiac grafts from prolonged ischemia reperfusion injury during HT. MircFoxo3 also indirectly affects miR-433 and miR-136 expression [70]. In addition, many ncRNAs can be used as noninvasive detection indicators, such as for the assessment of myocardial injury after HT. MiR-133b has been found to be an important marker of myocardial injury and to be associated with hemodynamic changes evident early after transplantation [34]. Circ-FSCN1 silencing produced tolerogenic dendritic cells, which prevented alloimmune rejection in HT, prolonged patient survival and reduced myocardial fibrosis [71]. However, whether functional interventions based on specific ncRNAs can block the development of long-term fibrosis after HT and thus improve survival after transplantation still needs to be explored in the future.

## 6. Conclusions and Perspectives

With the rapid development of the bioinformatics technologies, such as RNA sequencing and genomics, the role of ncRNAs in cardiovascular diseases has been extensively studied. NcRNAs are important regulators of cardiovascular disease through transcriptional regulation, posttranscriptional regulation and epigenetic level regulation of gene expression, especially in relation to the development of myocardial fibrosis. The construction of a cardiac fibrosis-specific ceRNA regulatory network could help further elucidate the molecular mechanism of the cardiac fibrosis process and provide plausible target genes for future research in this field. However, due to the limitations of the current experimental technologies, ceRNA regulatory networks are mainly confirmed by bioinformatics techniques to confirm their molecular mechanisms, large sample of population studies are still needed to explore the diagnosis, target therapy and prognosis of cardiovascular diseases. And the further study also is needed to confirm the molecular mechanisms of ncRNAs by constructing animal and cellular models of cardiovascular diseases. In addition, interspecies variability makes it more difficult to explore the the mechanisms of action of homologous ncRNAs through *in vivo* experiments. Finally, there is lack of a ceRNA network that can show regulator actions between coding RNAs and non-coding RNAs.
